# Incidental Cardiovascular Abnormalities in the Abdominal Aortic Aneurysm (AAA) Surveillance Population During the AAA Get Fit Trial: A Case Series and Review of the Literature

**DOI:** 10.7759/cureus.48271

**Published:** 2023-11-04

**Authors:** David J Flaherty, Adam Haque

**Affiliations:** 1 Trauma and Orthopaedics, Wythenshawe Hospital, Manchester, GBR; 2 Vascular Surgery, University of Manchester, Manchester, GBR

**Keywords:** major adverse cardiovascular events, opportunistic screening, aaa surveillance, cardiopulmonary exercise testing, abdominal aortic aneurysm

## Abstract

Background

The prevalence of cardiovascular disease and incidence of major adverse cardiovascular events (MACEs) is very high among the abdominal aortic aneurysm (AAA) surveillance population. Formal assessments of and interventions to reduce cardiovascular risk are not a routine part of the surveillance programme at present. However, its potential importance is highlighted by incidental findings during the AAA Get Fit Trial, a randomised controlled trial which included baseline cardiopulmonary exercise testing (CPET). We speculate that CPET can act as an opportunistic screening programme to identify cardiovascular disease in AAA surveillance patients.

Methods

The AAA Get Fit Trial was a prospective, randomised controlled trial at a tertiary vascular centre, Manchester University NHS Foundation Trust, conducted between November 2017 and August 2019. Patients underwent CPET at baseline, 8, 16, 24 and 36 weeks as well as clinical history and examination and blood tests. We report on incidental cardiovascular abnormalities diagnosed during the trial.

Results

Of the 59 participants in the trial, four (6.8%) were identified to have abnormal findings suggestive of unstable cardiovascular disease. On subsequent further investigation, two patients were diagnosed and treated for severe coronary artery disease after abnormal ECG findings were noted during CPET. One patient was diagnosed with unstable angina after obtaining a detailed history on baseline assessment which was treated medically before going on to have a successful elective AAA repair.

Conclusions

There is a high incidence of MACEs among this high-risk population both pre and perioperatively. Identifying and treating cardiovascular disease among the AAA surveillance population must be a focus of the future AAA screening programme.

## Introduction

Abdominal aortic aneurysm (AAA) affects more than one in 20 men over the age of 65 and causes 9,000 deaths annually in the UK [1,2]. Premature death in AAA is related to rupture which has an 80% mortality rate, and so national screening has been established to detect AAA before it reaches a size which significantly increases the risk of rupture [3]. As most of the AAAs discovered incidentally or through screening are below the threshold for rupture, a large population of patients are enrolled in surveillance programmes.

Although the pathophysiology of AAA is not classically atherosclerotic in nature, it is classed as a cardiovascular disease and shares risk factors with other atherosclerotic cardiovascular diseases [4]. As such, the co-existing prevalence of ischaemic heart disease (IHD) in AAA is 39%, with patients 72% more likely to suffer from IHD than an age-matched population [5]. Moreover, on analysis of a surveillance population in a screening programme, death due to cardiovascular disease was more likely than operative repair of the AAA [6]. Further, the UK small aneurysm trial found that almost 25% of screening patients not yet eligible for surgery had died at their follow-up as a result of cardiovascular or cerebrovascular disease [7]. These studies highlight both the high incidence of cardiovascular events among this population and its significant associated mortality [6,8]. Despite this, formal assessments of or interventions to reduce cardiovascular risk are not a routine part of surveillance programmes. In fact, evidence shows that both blood pressure and cholesterol increase, and cardiovascular fitness decreases, after entering surveillance [9]. Management of this increased cardiovascular risk within surveillance populations does not appear adequate with patients no more likely to be on primary preventative medication after they join AAA surveillance [9]. It has been demonstrated that patients undergoing major vascular surgery, such as AAA repair, have an increased risk of perioperative major adverse cardiovascular events (MACEs) [10]. The high risk of MACEs is due to a combination of the patient’s multiple medical co-morbidities and the operative stress response and subsequent raised catecholamine and cortisol levels [10]. Identifying and managing risk factors which increase MACEs should be a focus of management for patients with AAA.

Cardiovascular fitness before surgery can be measured using cardiopulmonary exercise testing (CPET), the parameters of which have been shown to be associated with perioperative and long-term survival after AAA repair [11]. Favourable CPET parameters have also demonstrated a reduced cardiovascular risk, and the test itself can detect and stratify cardiovascular disease [12,13]. In particular, decreased peak oxygen consumption (peak VO_2_) and a low or plateauing oxygen pulse during exercise can indicate cardiac abnormalities warranting further investigation [12-14]. Patients also have a full exercise electrocardiogram (ECG) during CPET which can detect exercise-induced ischaemia or arrhythmias. As CPET is performed to maximal exertion, this is equivalent to a standard exercise stress ECG.

Identifying incidental cardiovascular issues through CPET for AAA patients is considered a form of opportunistic screening. Typically, opportunistic screening involves identifying incidental findings while performing formal investigations such as a CT scan [15]. AAAs themselves are commonly first identified during opportunistic screening CT scans, requested to investigate alternative pathologies [16-18]. However, opportunistic screening can also include identifying co-existing pathology while performing routine investigations for AAA patients as part of the surveillance programme. One such study identified co-existing pathology and risk factors in patients diagnosed with a small AAA (<5.5 cm), highlighting that this cohort had high rates of hypertension (57.8%), diabetes mellitus (17.7%), and hypercholesterolaemia (53%) [9]. Moreover, Saratzis et al. (2017) noted that only a small percentage of patients were taking either aspirin and/or clopidogrel, and a relatively small proportion had been prescribed a statin (60.9%) [9]. This example of opportunistic screening identified multiple potential targets to reduce the risk of cardiovascular disease for patients in the AAA surveillance cohort [9]. Moreover, we can also consider the role of opportunistic screening to identify mental health issues in patients on the AAA screening programme. Evidence has demonstrated that mental quality of life scores reduce once patient’s AAAs are identified [19]. To our knowledge, this is the first study to highlight the benefits of opportunistic screening with the use of formal investigations to identify cardiovascular disease in known AAA patients [20]. The AAA Get Fit Trial was a randomised controlled trial (RCT) of patient-directed community-based exercise to improve the CPET parameters of fitness associated with increased perioperative and long-term risk following elective AAA repair [21]. CPET, which can discover previously undiagnosed abnormalities, was performed as part of baseline assessment and assessments at 8, 16, 24, and 36 weeks. We report a case series of four patients in whom incidental, but serious, cardiovascular abnormalities were detected. We provide a detailed explanation of the diagnosis and management of each cardiovascular abnormality detected in the AAA surveillance patients. Identifying and treating cardiovascular disease in this high-risk population may reduce perioperative mortality and specifically the occurrence of MACEs during surveillance.

## Materials and methods

The AAA Get Fit Trial was a prospective RCT at a tertiary vascular centre, Manchester University NHS Foundation Trust, conducted between November 2017 and August 2019.

Participants

Consecutive, eligible, and willing participants were recruited from the AAA surveillance clinic at MFT Wythenshawe based on the inclusion and exclusion criteria.

Inclusion Criteria

We included individuals who met the following inclusion criteria: (1) men with AAA ≥ 3.0 < 5.0 cm and women with AAA ≥ 3.0 < 4.5 cm; (2) individuals potentially fit for elective AAA repair (open or endovascular aneurysm repair (EVAR)); (3) those aged 60-85 years inclusive; and (4) those willing and able to complete CPET and engage in gym and/or home-based exercise training.

Exclusion Criteria

We excluded individuals who met the following exclusion criteria: (1) patients deemed not fit for elective AAA repair (open or EVAR) even following exercise training and weight loss; (2) those unable or unwilling to undertake CPET or exercise training; (3) individuals with severe liver disease (international normalized ratio >2, serum albumin <3.0 g/dL, bilirubin >50 µmol/L); (4) those with unstable angina occurring more than once daily, angina that was increasing in frequency or precipitated by less exertion, angina at rest, or of recent onset (<2 months); (5) those with uncontrolled (heart rate (HR) >90 beats/minute) atrial fibrillation (AF) or other arrhythmias (untreated paroxysmal AF); (6) individuals with moderate or severe aortic valve stenosis (peak systolic pressure gradient >40 mmHg or with an aortic valve area <1 cm^2^); (7) individuals with pericarditis or myocarditis within the previous six months; (8) those with >2 mm ST depression during baseline assessment CPET; and (9) those diagnosed or being treated for a malignancy, other than basal cell carcinoma, within the previous 12 months.

To fully assess eligibility, consenting patients underwent baseline assessment which included a full discussion of the protocol, signing of the consent form, recording of a detailed past medical history, clinical examination, phlebotomy for cardiovascular biomarkers, and a full CPET test. Results from this assessment also allowed subsequent randomisation which included minimisation for the important CPET parameter and primary outcome of peak VO_2_.

Cardiopulmonary exercise testing

All CPET studies were performed according to the same 15 W ramp test (Wasserman) protocol, performed on cycle ergometry [22]. Studies were performed using the Ultima™ CardiO2® MedGraphics gas exchange analysis system linked to the BreezeSuite™ software package (Medical Graphics, St Paul, MN, USA), which was maintained and serviced accordingly. Predicted values were calculated automatically by the in-built software using the validated and reliable Wasserman equations [23,24]. The equipment was manually calibrated before each CPET session as per the manufacturer’s instructions for use. A 12-lead wireless Bluetooth ECG was also connected to the BreezeSuite™ software during each test. Elevation or depression of the ST segment of more than 2 mm was considered pathological. Each CPET was performed to maximal exertion, as determined by the patient unless significant abnormalities were detected, in which case the test would be stopped for safety reasons. All CPETs were performed and interpreted by a trained research doctor accredited in clinical CPET performing and reporting by the Perioperative Exercise Testing and Training Society (POETTS). A sample of these was cross-checked to ensure accuracy by a similarly accredited physician.

Interventions

Patients were randomly assigned to either 24-week, patient-directed, community-based exercise or control which consisted of standard advice only. Further details on these interventions are described in the write-up of the full trial [21].

Outcomes

Outcome measures were recorded at 8, 16, 24 and 36 weeks from baseline assessment. A full CPET was performed at each of these time intervals as the CPET parameters of peak VO_2_, anaerobic threshold, ventilatory equivalent for carbon dioxide at the anaerobic threshold, and work at peak VO_2_ were all outcome measures. Other outcome measures included cardiovascular risk biomarkers, anthropometric markers of cardiovascular risk, health-related quality of life, and habitual physical activity. Further details on this, sample sizes, randomisation, blinding, and statistical methods are provided in the write-up of the trial [21].

## Results

Between November 2017 and August 2019, In accordance with the power calculation for our primary outcome of peak VO_2_, 59 patients were recruited into the AAA Get Fit Trial. Overall, four of the 59 patients (6.8%) presented abnormal findings in the trial requiring further investigation. Three of the patients presented immediate abnormal findings on baseline assessment. One patient completed the full trial but their CPET findings were unsatisfactory requiring further medical investigation and intervention. The results outline the findings and management of these four patients identified during opportunistic screening.

Case one

**Figure 1:**
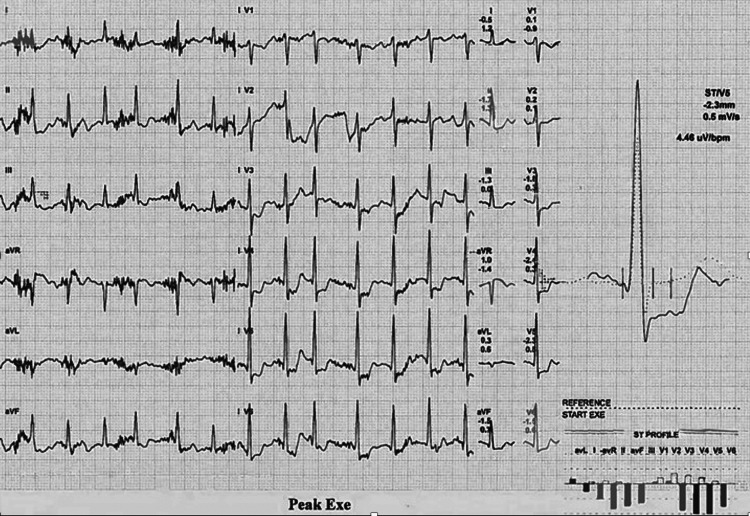
CPET ECG of case one at peak exercise demonstrating pathological ST depression in the anterolateral leads. CPET: cardiopulmonary exercise testing; ECG: electrocardiogram

Case one involved a 76-year-old retired Senior Lecturer who was on the surveillance programme for a 4.6 cm AAA. He had a history of IHD having had a myocardial infarction (MI) in 1999 which was managed conservatively with the best medical therapy. His other past medical history included pre-diabetes, peripheral arterial disease (PAD), chronic kidney disease, osteoarthritis, and oesophagitis. Detailed history taking during baseline assessment of the AAA Get Fit Trial did not reveal any significant, current symptoms suggestive of cardiac ischaemia. However, during CPET testing significant ST depression was noted in the anterolateral leads, maximally resulting in a 3.2 mm depression near the end of the exercise (Figure 1).

Despite the abnormal ECG, the patient did not report any symptoms during testing, though in recovery he did note a ‘tingly’ left hand. He was subsequently referred to cardiology on an urgent basis who performed a transoesophageal echocardiogram (TOE). The TOE demonstrated a normal-sized left ventricle with normal systolic function and no detectable valvular pathology. However, due to the results of the CPET, the patient underwent a stress echocardiogram which was positive for ischaemia in six segments of the left anterior descending artery (LAD) territory, accounting for a third of the myocardium. Based on these findings, a diagnostic coronary angiogram was performed which showed a long segment of calcified severe atherosclerotic disease in the proximal to mid-LAD and occlusion of the obtuse marginal artery (OM). Due to the length and distribution of the disease, the patient underwent coronary artery bypass grafting (CABG), including bypass of the LAD with the left internal mammary artery (LIMA) and bypass of the OM with the long saphenous vein (LSV). Postoperatively, the patient’s care was unremarkable. He was followed up in the cardiology outpatient department having made an excellent recovery and attended regular cardiac rehabilitation classes. The patient has subsequently undergone a successful open AAA repair with no MACEs during the peri or postoperative period.

Case two

**Figure 2:**
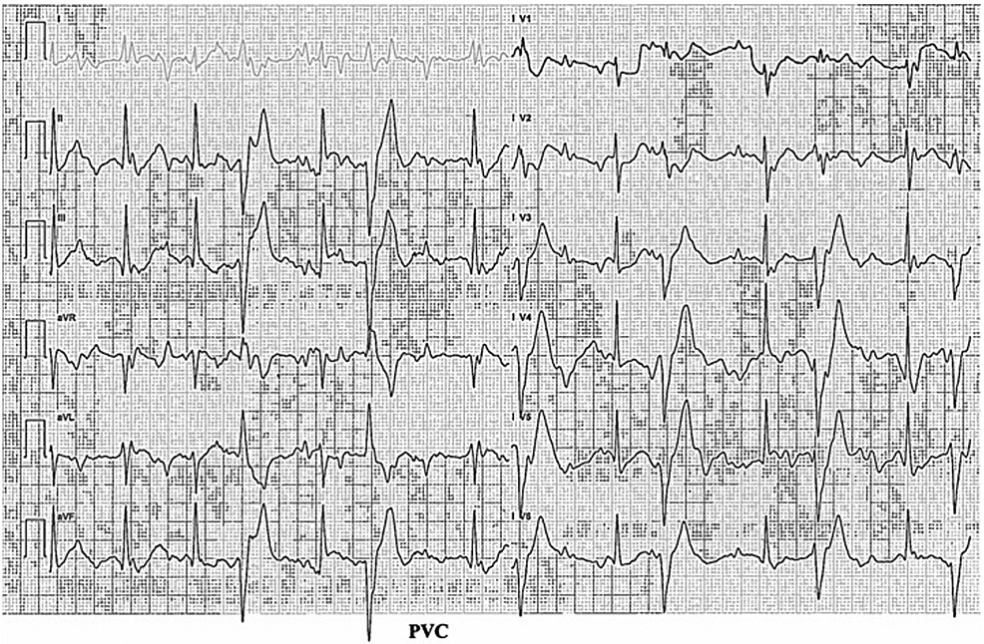
CPET ECG of case two demonstrating multiple ventricular ectopics within the ECG rhythm. CPET: cardiopulmonary exercise testing; ECG: electrocardiogram

Case two was a 74-year-old male in surveillance for a 4.5 cm AAA. The patient was known to have IHD and had undergone two previous CABG operations in 1987 and 1992. His past medical history included PAD, COPD, and Crohn’s disease. He was a current smoker. On CPET, during baseline assessment, the ECG demonstrated normal sinus rhythm at rest. However, very early on in exercise (41 watts/2 minutes 44 seconds), despite being asymptomatic, the ECG demonstrated multiple and frequent ventricular ectopic beats (Figure 2). The test was stopped, and the rhythm returned to normal in recovery.

Based on these findings, the patient was referred to cardiology who initially performed a TOE which showed mildly impaired left ventricular function. This was followed by a coronary angiogram which showed severe native triple-vessel disease, a patent LIMA graft to the LAD, but an almost occluded LSV graft to the right coronary artery (RCA) and a totally occluded LSV graft to the OM artery. As a result, there was only one patent graft supplying the myocardium. There was potential to improve myocardial blood flow through percutaneous coronary intervention (PCI), but due to the complexity of the disease, this procedure carried a significant risk of major adverse events, including MI and stroke. At the time of writing, the patient is still considering whether to proceed with further coronary artery intervention. He is still attending surveillance for his AAA which has now reached 4.8 cm.

Case three

**Figure 3:**
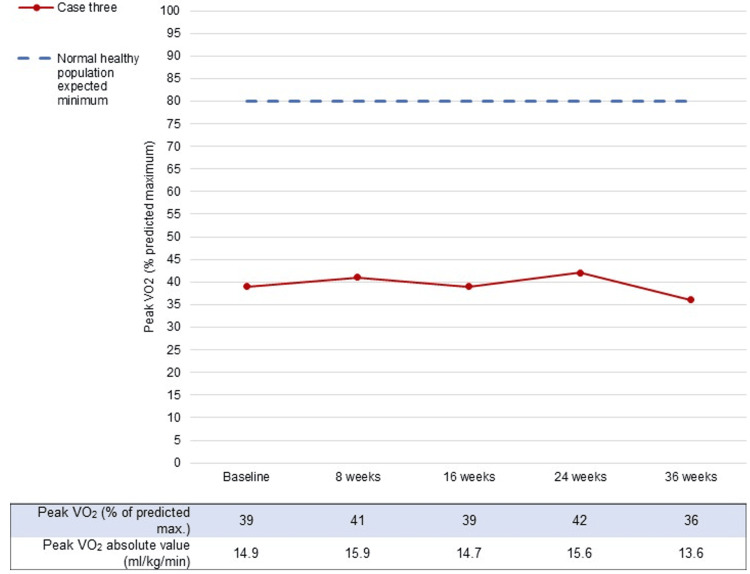
Changes in peak VO2 in case three during the AAA Get Fit Trial.

Case three was a 61-year-old male on surveillance with a 3.6 cm AAA. His past medical history included PAD, hypertension, and hypercholesterolaemia. He was an ex-smoker. Despite adhering to the exercise programme, his improvements in CPET parameters were minimal, with a peak VO_2_ never reaching more than 42% of what would be expected for his age, height, weight, gender, and ethnicity. His absolute peak VO_2_ (<15 mL/kg/minute) would have made him a high risk for operative intervention at three of the time intervals, including the one recorded at the final follow-up (Figure 3). These changes were incongruous with the overall mean (95% CI) improvements in the study for the intervention group which demonstrated an increase of at least 2.0 mL/kg/minute at all time intervals from 16 weeks onwards.

**Figure 4:**
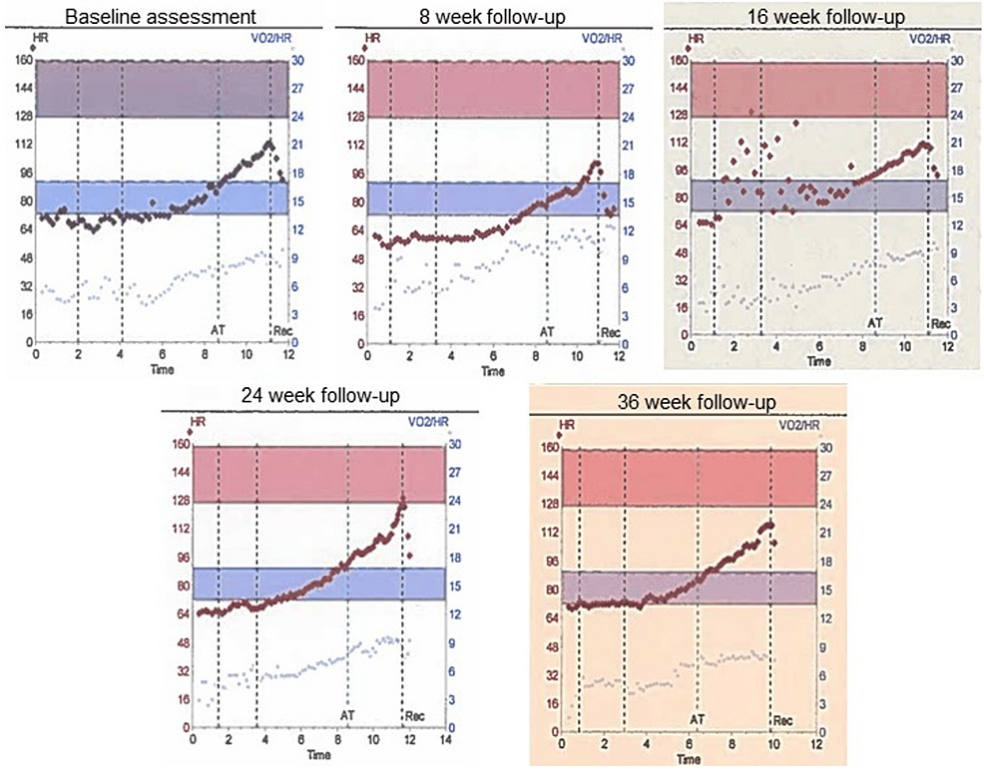
Oxygen pulse of case three at all time points during the trial. The oxygen pulse is represented in each graph by the blue/purple hollow circles in the lower section of each graph. The blue/purple band is the expected range in a normal individual with the same demographics. The graphs demonstrate an overall low oxygen pulse with an early plateau.

The lack of improvement in CPET parameters and the potential underlying diagnosis could also be appreciated by reviewing the oxygen pulse. Oxygen pulse is the amount of oxygen that the lungs transfer into the circulatory system with each heartbeat and can be roughly equated to cardiac output. Cardiac output is the product of stroke volume and heart rate, thus oxygen pulse can be considered a surrogate for stroke volume. A normal oxygen pulse, like stroke volume, should increase during exercise and then start to tail off towards maximal exertion when any increase in cardiac output is a result of an increasing heart rate. In people who have an underlying functional cardiac abnormality, this normal response does not occur, as the stroke volume is unable to increase, and so oxygen pulse either does not change at all throughout the test or plateaus early in testing. For case three, his oxygen pulse was low throughout testing, never achieving even the lower threshold of his predicted parameters. An early plateau can be clearly seen at baseline assessment, eight-week follow-up, and 36-week follow-up and to a lesser extent at 16 and 24-week follow-ups (Figure 4).

It is possible the oxygen pulse remained low due to low effort from the patient during testing. However, as there was repeat testing, for an individual who appeared motivated to remain in a trial for nine months, it would be unusual to display a complete lack of motivation and effort during the testing itself. As a result of this testing, the patient was offered further investigation, with TOE in the first instance, to further assess his cardiac function. At present, the patient has refused further investigation but does remain on the standard AAA surveillance programme.

Case four

Case four was a 63-year-old father of 15 who was recruited from surveillance with a 4.6 cm AAA. He had a significant past medical history of left ventricular systolic dysfunction, MI, obesity, COPD, hypertension, and osteoarthritis. He was an active smoker. During detailed history taking at baseline assessment, he revealed he had intermittently been feeling tightness in his chest at rest which intensified on minimal exertion. These symptoms were pathognomonic for unstable angina and, as per POETTS guidelines, CPET was not attempted. The patient was then referred to cardiology who optimised his medical management.

Six months later, the patient’s AAA had increased in size to 6.6 cm likely due to an underlying inflammatory process. TOE at this time revealed moderate left ventricular dilatation with severely impaired left ventricular and right atrial systolic dysfunction. In preparation for a likely urgent AAA repair, he had a preoperative CPET which revealed him to be at significant risk for open repair, with an estimated perioperative mortality of 5-10%. Despite this, the patient opted for open surgical repair which was performed as a dual-consultant case. He had an uneventful three-day stay in the Intensive Care Unit and was subsequently discharged on day eight. He was re-admitted for a washout of a seroma in the right groin a month later, but apart from this, he had an excellent postoperative recovery.

## Discussion

Of the 59 AAA patients, four (6.8%) entering the AAA Get Fit Trial demonstrated cardiovascular abnormalities which required further investigation and/or treatment. The cardiovascular abnormalities were identified at various points of the screening programme; one during baseline history taking, two patients on ECG analysis while undergoing CPET, and one patient during post-trial analysis of CPET parameters. Substantial evidence in the literature has demonstrated the high incidence of MACEs in AAA patients both pre and perioperatively [10,25]. We present the first study which demonstrates in specific detail how these cardiovascular abnormalities identified were investigated and subsequently treated.

Two of the patients were diagnosed with severe coronary artery disease after abnormal ECG analysis during CPET, requiring medical and surgical intervention. One patient demonstrated signs and symptoms of unstable angina during the initial history taking and examination. The patient’s angina was managed medically before a successful AAA elective repair. The final patient demonstrated signs of cardiovascular disease during CPET analysis but has thus far refused further investigations. Cardiovascular abnormalities identified during CPET testing in the AAA Get Fit Trial may not have been discovered if patients were not enrolled in the trial. The incidence of MACEs in AAA patients is extremely high both preoperatively and perioperatively [10,25]. As a direct result of the trial protocol which included extensive history taking, basic investigations such as ECGs, and formal exercise testing, significant cardiovascular abnormalities were identified preventing MACEs. By identifying patients with cardiovascular disease early, there is the possibility of preventing a large number of MACEs and reducing mortality significantly for this high-risk population on the AAA screening programme, particularly for those undergoing AAA repair.

Moreover, we believe this exercise and screening programme involving CPET may also prove to be cost-effective. The AAA screening programme itself, which offers an ultrasound scan for males >65 years of age is considered to be cost-effective [26]. This is based on the estimate that 667 men need to be screened to prevent one premature AAA-related death [26]. If we consider that the prevalence of MACEs in unrepaired AAA patients is as high as 38% over five years and that our screening programme identified cardiovascular pathology in 5.1% of patients, a CPET screening programme may possibly play a cost-effective role for AAA patients [25]. Furthermore, it has been estimated in 2016 that a patient’s first MI will cost the NHS £4,275 with those requiring angioplasty or a CABG costing a further £5,635 [27]. In contrast, an outpatient CPET test has an NHS tariff in 2023 of £284 [28]. Although we acknowledge a formal cost analysis if required to make a conclusion, we suggest that CPET testing as part of the AAA screening programme may demonstrate cost-effectiveness.

CPET may demonstrate a role as part of an opportunistic screening programme for cardiovascular disease in AAA patients. However, along with identifying cardiovascular disease, CPET has multiple uses for patients on the surveillance programme, including identifying patients suitable for elective AAA repair [21]. Literature has previously demonstrated that CPET can act as a predictor of mortality and morbidity in AAA repair and can be used as part of the decision-making process when determining a patient’s suitability for operative management [29]. Further, patients with a low CPET score have the potential to enter an exercise programme, as demonstrated in the AAA Get Fit Trial. This can act as a ‘pre-habilitation’ programme which has been demonstrated to reduce all cardiovascular-related mortality regardless of whether the patients undergo surgical repair [21]. Therefore, irrespective of whether CPET identifies cardiovascular disease, it has the potential to form an integral part of the AAA surveillance programme.

We recognise the limitations of this study, in particular regarding the small number of patients recruited into the trial. Although we have reported the incidence of cardiovascular abnormalities among only 59 patients, the high prevalence of abnormal findings requiring investigation (6.8%) emphasises the importance of expanding this study. Moreover, only 59 of 154 eligible patients agreed to join the original trial, which may be due to the trial’s requirement for regular community-based exercise and multiple face-to-face reviews. It is possible that with a less invasive screening method such as a single CPET, stress ECG, and baseline history and examination, a large number of cardiovascular abnormalities can still be identified among this high-risk population.

## Conclusions

Identifying and treating cardiovascular disease among the AAA surveillance population must be a focus of the future AAA screening programme. There is a high incidence of MACEs among this high-risk population pre and perioperatively. This study has demonstrated how this exercise programme including CPET testing can identify cardiac disease followed by investigation and treatment reducing mortality for AAA surveillance patients. More research is required to identify the most cost-effective and appropriate method of identifying cardiac disease with a suitable pathway set up to manage patients accordingly.

## References

[1] Reimerink JJ, van der Laan MJ, Koelemay MJ, Balm R, Legemate DA (2013). Systematic review and meta-analysis of population-based mortality from ruptured abdominal aortic aneurysm. Br J Surg.

[2] Stather PW, Sidloff DA, Rhema IA, Choke E, Bown MJ, Sayers RD (2014). A review of current reporting of abdominal aortic aneurysm mortality and prevalence in the literature. Eur J Vasc Endovasc Surg.

[3] (1998). Mortality results for randomised controlled trial of early elective surgery or ultrasonographic surveillance for small abdominal aortic aneurysms. The UK Small Aneurysm Trial Participants. Lancet.

[4] Ailawadi G, Eliason JL, Upchurch GR Jr (2003). Current concepts in the pathogenesis of abdominal aortic aneurysm. J Vasc Surg.

[5] Kent KC, Zwolak RM, Egorova NN (2010). Analysis of risk factors for abdominal aortic aneurysm in a cohort of more than 3 million individuals. J Vasc Surg.

[6] Haque A, McCollum C (2022). Patients on AAA surveillance are at greater threat of cardiovascular events or malignancy than their AAA: outcomes of AAA surveillance over 19 years at a tertiary vascular centre. Ann Vasc Surg.

[7] Lederle FA, Wilson SE, Johnson GR (2002). Immediate repair compared with surveillance of small abdominal aortic aneurysms. N Engl J Med.

[8] Grant SW, Hickey GL, Grayson AD, Mitchell DC, McCollum CN (2013). National risk prediction model for elective abdominal aortic aneurysm repair. Br J Surg.

[9] Saratzis A, Sidloff D, Bown MJ (2017). Cardiovascular risk in patients with small abdominal aortic aneurysms. Lancet.

[10] Lee C, Columbo JA, Stone DH, Creager MA, Henkin S (2022). Preoperative evaluation and perioperative management of patients undergoing major vascular surgery. Vasc Med.

[11] Grant SW, Hickey GL, Wisely NA (2015). Cardiopulmonary exercise testing and survival after elective abdominal aortic aneurysm repair. Br J Anaesth.

[12] Swank AM, Horton J, Fleg JL (2012). Modest increase in peak VO2 is related to better clinical outcomes in chronic heart failure patients: results from heart failure and a controlled trial to investigate outcomes of exercise training. Circ Heart Fail.

[13] Keteyian SJ, Patel M, Kraus WE (2016). Variables measured during cardiopulmonary exercise testing as predictors of mortality in chronic systolic heart failure. J Am Coll Cardiol.

[14] Kinnear W, Blakey J (2014). A Practical Guide to the Interpretation of Cardiopulmonary Exercise Testing.

[15] Jacobs PC, Mali WP, Grobbee DE, van der Graaf Y (2008). Prevalence of incidental findings in computed tomographic screening of the chest: a systematic review. J Comput Assist Tomogr.

[16] Tisi PV, McLain AD, Jeddy TA, Ashton HA, Scott RA (1998). Screening for abdominal aortic aneurysm: is opportunistic detection a realistic alternative?. Eur J Vasc Endovasc Surg.

[17] Claridge R, Arnold S, Morrison N, van Rij AM (2017). Measuring abdominal aortic diameters in routine abdominal computed tomography scans and implications for abdominal aortic aneurysm screening. J Vasc Surg.

[18] Kodenko MR, Vasilev YA, Vladzymyrskyy AV (2022). Diagnostic accuracy of AI for opportunistic screening of abdominal aortic aneurysm in CT: a systematic review and narrative synthesis. Diagnostics (Basel).

[19] Bath MF, Sidloff D, Saratzis A, Bown MJ (2018). Impact of abdominal aortic aneurysm screening on quality of life. Br J Surg.

[20] Chan WK, Yong E, Hong Q (2021). Systematic review and meta-analysis of the prevalence of abdominal aortic aneurysm in Asian populations. J Vasc Surg.

[21] Haque A, Wisely N, McCollum C (2022). Editor's choice - the Abdominal Aortic Aneurysm Get Fit Trial: a randomised controlled trial of exercise to improve fitness in patients with abdominal aortic aneurysm. Eur J Vasc Endovasc Surg.

[22] Wasserman K, Hansen JE, Sue DY, Stringer WW, Whipp BJ (2005). Principles of Exercise Testing and Interpretation: Pathophysiology and Clinical Applications.

[23] Arena R, Myers J, Abella J (2009). Determining the preferred percent-predicted equation for peak oxygen consumption in patients with heart failure. Circ Heart Fail.

[24] Wasserman K, Hansen JE, Sue DY (2011). Principles of Exercise Testing and Interpretation: Including Pathophysiology and Clinical Applications. Fifth Edition.

[25] Nastasi DR, Norman R, Moxon JV, Quigley F, Velu R, Jenkins J, Golledge J (2021). The potential benefits and costs of an intensified approach to low density lipoprotein cholesterol lowering in people with abdominal aortic aneurysm. Eur J Vasc Endovasc Surg.

[26] Wanhainen A, Hultgren R, Linné A (2016). Outcome of the Swedish nationwide abdominal aortic aneurysm screening program. Circulation.

[27] Danese MD, Gleeson M, Kutikova L (2016). Estimating the economic burden of cardiovascular events in patients receiving lipid-modifying therapy in the UK. BMJ Open.

[28] (2023). NHS England National Tariff. https://www.england.nhs.uk/pay-syst/national-tariff.

[29] Thompson AR, Peters N, Lovegrove RE, Ledwidge S, Kitching A, Magee TR, Galland RB (2011). Cardiopulmonary exercise testing provides a predictive tool for early and late outcomes in abdominal aortic aneurysm patients. Ann R Coll Surg Engl.

